# Thoracic gas compression during forced expiration in patients with emphysema, interstitial lung disease and obesity

**DOI:** 10.1186/1471-2466-14-34

**Published:** 2014-03-05

**Authors:** Päivi L Piirilä, Ulla Hodgson, Tomi Wuorimaa, Hans-Jürgen Smith, Anssi RA Sovijärvi

**Affiliations:** 1 Unit of Clinical Physiology, Department of Clinical Physiology and Nuclear Medicine, HUS Medical Imaging Center, Helsinki University Central Hospital, P.O.Box 340, Helsinki, HUS 00029, Finland; 2 Heart and Lung Center Helsinki, Helsinki University Central Hospital, Helsinki, Finland; 3 Research in Respiratory Diagnostics, Bahrendorfer Str. 3, Berlin 12555, Germany

**Keywords:** Chronic obstructive pulmonary disease, Emphysema, Flow plethysmography, Healthy control, Interstitial lung disease, Obesity, Thoracic gas compression

## Abstract

**Background:**

Dynamic gas compression during forced expiration has an influence on conventional flow-volume spirometry results. The extent of gas compression in different pulmonary disorders remains obscure. Utilizing a flow plethysmograph we determined the difference between thoracic and mouth flows during forced expiration as an indication of thoracic gas compression in subjects with different pulmonary diseases characterized by limitations in pulmonary mechanics.

**Methods:**

Patients with emphysema (N = 16), interstitial lung disease (ILD) (N = 15), obesity (N = 15) and healthy controls (N = 16) were included. Compressed expiratory flow-volume curves (at mouth) and corresponding compression-free curves (thoracic) were recorded. Peak flow (PEF) and maximal flows at 75%, 50% and 25% of remaining forced vital capacity (MEF75, MEF50 and MEF25) were derived from both recordings. Their respective difference was assessed as an indicator of gas compression.

**Results:**

In all groups, significant differences between thoracic and mouth flows were found at MEF50 (p < 0.01). In controls, a significant difference was also measured at MEF75 (p <0.005), in emphysema subjects, at PEF and MEF75 (p < 0.05, p < 0.005) and in obese subjects at MEF75 (p <0.005) and MEF25 (p < 0.01). ILD patients showed the lowest difference between thoracic and mouth flows at MEF75 relative to controls and emphysema patients (p < 0.005, p < 0.001). Obese subjects did not differ from controls, however, the difference between thoracic and mouth flows was significantly higher than in patients with emphysema at MEF50 (p < 0.001) and MEF25 (p < 0.005).

**Conclusions:**

Alveolar gas compression distorts the forced expiratory flow volume curve in all studied groups at the middle fraction of forced expiratory flow. Consequently, mouth flows are underestimated and the reduction of flow measured at 75% and 50% of vital capacity is often considerable. However, gas compression profiles in stiff lungs, in patients with decreased elastic recoil in emphysema and in obesity differ; the difference between thoracic and mouth flows in forced expiration was minimal in ILD at the first part of forced expiration and was higher in obesity than in emphysema at the middle and last parts of forced expiration.

## Background

During forced expiration alveolar gas is compressed due to muscular force and the combined effect of elastic pressures of the thorax and lungs acting against airway resistances [1]. The compression-free flow-volume curve can be measured by a flow plethysmograph (transmural plethysmograph), employed in a constant pressure, pressure-corrected mode, by integrating air flow through a pneumotachograph in the wall of a body plethysmograph. A flow plethysmograph measures the volume displacement of the chest movement simultaneously with the spirometric measurement of integrated flow at the mouth. This method permits accurate measurements of changes in thoracic gas volume (TGV) during forced expiratory manoeuvres [2,3].

Several reports exist on increased thoracic gas compression in asthma, the compression increasing with increasing airways resistance [4-6]. Alveolar gas compression during forced expiration causes a considerable artifact on flow-volume curves derived from mouth flow [7-9]. This divergence is also seen in flow-volume loops during bronchodilation testing, especially in the mid-range of forced expiratory flow [5,6]. Elevated gas compression at the mid-fraction of the forced expiratory flow-volume loop has been reported for smoking subjects [5] and in chronic obstructive pulmonary disease (COPD) [10,11]. However, as far as we know, no data are available on gas compression in interstitial lung disease (ILD) or obesity.

Gas compression can modify the results of conventional forced spirometry. Therefore, we were interested in studying gas compression in ILD and obesity relative to healthy controls and patients with emphysema.

Thoracic gas compression was investigated in four groups of subjects with different pulmonary mechanics: healthy non-smoking subjects (controls), healthy non-smoking obese subjects (obese), patients with interstitial lung disease (ILD) and patients with emphysema (emphysema).

### Patients

The anthropometric data and smoking habits of subjects included in the study are summarized in Table 1. Healthy non-smoking subjects (N = 16) (BMI <30), healthy obese non-smoking subjects (N = 15) (BMI >30), non-smoking patients with interstitial lung disease (ILD) (N = 15) (BMI <30) and patients with emphysema (N = 16) (BMI < 30, FEV1/FVC < 0.7) were investigated. However, patients with a significant bronchodilator response (delta FEV1 ≥ 12% and >200 mL) [12] in spirometry were excluded. The group of patients with ILD (N = 15) consisted of 6 patients with idiopathic pulmonary fibrosis (IPF), diagnosed according to the ATS/ERS/JRS/ALAT 2011 guidelines [13]; 4 patients with non-specific interstitial pneumonia (NSIP)/IPF-type ILD related to a connective tissue disorder (1 with non-specified collagenosis, 1 with mixed type of collagenosis, 1 with scleroderma and 1 with rheumatoid arthritis); 3 patients with sarcoidosis, 1 patient with fibrotic NSIP, and 1 patient with allergic alveolitis.

**Table 1:** Anthropometric data and smoking history of patients and controls (mean (SD) and (range))

	**Controls, N = 16**	**ILD, N = 15**	**Emphysema, N =16**	**Obese, N = 15**
Men/women	10/6	7/8	10/6	4/11
Weight (kg)	70.94 (9.54) (51 – 88)	71.65 (9.1) (52–85)	58.4 (13.7) (35–83)	95.01 (16.8) (78 – 134)
Height (cm)	170.69 (6.05) (161 – 179)	169.0 (9.6) (150 – 185)	168.9 (9.1) (155–183)	163.1 (9.2) (148 – 181)
BMI (kg/m2)	24.18 (2.30) (19.7 – 27.5)	25.0 (1.83) (20.8 – 28.4)	20.3 (3.81) (12.7 – 27.1)	35.5 (3.1 ) (30.8 – 41.8)
Age (years)	65.06 (11.19) (35 – 79)	60.1 (11.1) (40 – 77)	62.7 (7.36) (44 – 74)	63.4 (9.2) (44 – 78)
Smokers/ex-smokers	0/0	0/1	10/5	0/0
Smoking (pack- years)	0	20	41.4 (8.0)	0

One patient with ILD was an ex-smoker with 20 pack-years who had stopped smoking 3 years earlier. He had no signs of obstruction according to the results of spirometry and conventional constant-volume body plethysmography. Other patients with ILD were lifelong non-smokers. Five patients used peroral prednisolone, 3 patients’ triple-therapy consisted of peroral prednisolone, azathioprine and N-acetylcysteine. One patient used methotrexate, 1 N-acetylcysteine, 1 cyclosporin and hydroxychloroquin, 1 peroral prednisolone and azathioprine, 1 N-acetylcysteine and peroral prednisolone, and 2 did not take any specific medication for their ILD.

The obese subjects (N = 15) had no pulmonary diseases, but 10 of them suffered from arterial hypertension treated with different antihypertensive drugs and 4 used diabetes medication.

The patients in the emphysema group showed a decreased diffusing capacity for carbon monoxide (< 74% of predicted value) confirming emphysema. With the mean value of 38.1% (SD 16.9) of predicted value [14], they suffered from moderate emphysema. Most of them were current smokers, but 5 had stopped smoking on average of 5.5 years earlier (Table 1). The patients with emphysema (N = 16) also fulfilled the international criteria for COPD [15]. They were smokers with symptoms of dyspnoea, their post-bronchodilation FEV1/FVC ratio was lower than 0.7 and their FEV1 was below 80% of predicted value [14]. According to a recent spirometric classification of COPD [16], 4 and 12 patients were classified as having moderate and severe COPD, respectively. Two patients took no medication; however, all others used a combination of long-acting beta-sympathomimetic and inhaled steroid (9 patients), tiotropium bromide monohydrate (6 patients), inhaled beclomethasone or budesonide (3 patients), peroral theophylline (4 patients), peroral prednisolone, (2 patients) and ipratropium bromide and/or salbutamol as a rescue medication (3 patients).

The study protocol was approved by the Ethics Committee of Helsinki University Central Hospital, and all participants gave their informed consent.

## Methods

Spirometry was measured with a Medikro SpiroStar spirometer (Medikro Kuopio, Finland) according to recent international guidelines [17]. Bronchodilation testing was performed using 0.4 mg of salbutamol aerosol (Ventoline®, Glaxo UK) given with a spacer. Post-bronchodilator spirometry was recorded 15 minutes after the salbutamol dose.

Diffusing capacity measurements were performed according to international guidelines [18]. Vital capacity (VC) was measured first, allowing standardization of the inspiratory volume to 90% of individual maximal VC [14]. The mean value of the parameters of two diffusing capacity measurements was presented as the result. Measurements were corrected for haemoglobin concentration.

The body plethysmographic measurements were performed first in conventional constant-volume mode (MasterScreen Body Version 4.3, Würzburg, Germany). The atmospheric pressure was measured using a Vaisala device (Vaisala, Finland). Flow-volume calibration according to the three-flow protocol was performed with a Jaeger 3 L calibration syringe. If the deviation of the registered volume was within ±3.5% for all flows, the calibration was regarded as successful. Box verification/calibration included two procedures. The time constant of the box was verified to lie within the range of 4–7 seconds. Box shift volume was calibrated following the recommendations of the manufacturer.

For flow plethysmography, corresponding calibration methods were applied. To allow flow-volume calibration of the spirometer connected to the box chamber, the box was set to the flow plethysmographic (transmural) mode by removing the closure on the box wall hole and closing the door of the box. The calibration pump strokes were directed through the box chamber pneumotachograph. A volume calibration was accepted if the stroke volumes did not exceed the ±3.5% range.

All investigations were performed in conventional constant volume mode first. The measurements of specific resistance breathing loops and functional residual capacity (FRCpleth) were performed with a panting frequency of 0.5 Hz [14]. The 4 second shutter manoeuvre (FRCpleth) was linked to a maximal spirometric vital capacity manoeuvre for determination of residual volume (RV) and total lung capacity (TLC). The system automatically derived total specific conductance (sGtot) from the breathing loops and determined total respiratory resistance (Rtot).

In flow plethysmographic mode, the uncompressed flow-volume curve at the chest wall and the compressed flow-volume loops at the mouth were measured simultaneously. The differences between compression-free or thoracic flows and corresponding compressed mouth flows were regarded as rough estimates of the degree of gas compression at levels of peak flow (PEF) and maximal expiratory flows at 75%, 50% and 25% of the remaining forced vital capacity (MEF75, MEF50 and MEF25). The volume reference was the same in both thoracic and mouth flow measurements, i.e. the forced vital capacity.

### Statistical methods

The intra-individual comparisons of thoracic and mouth flow at the levels of PEF, MEF75, MEF50 and MEF25 within the patient groups were investigated with paired *t*-test. Between the groups, gas compression (measured as the difference between thoracic and mouth flow at different volume levels) was evaluated utilizing the non-paired *t*-test. The comparisons of obese subjects with other groups were adjusted for gender and age, the other comparisons were adjusted for age and BMI. The significance level of p-value was assessed after Bonferroni correction.

## Results

The results of spirometry, diffusing capacity and constant volume body plethysmography for the patient groups are summarized in Table 2. In ILD, restrictive ventilatory impairments and reduction of diffusing capacity were found. Reduced FEV1/FVC ratio, increased Rtot and reduced sGtot were noted as signs of obstruction, and limited DLCOc and DLCOc/VA (KCOc) as signs of emphysema. In obesity, low ERV and FRCpleth were observed with a normal diffusing capacity.

**Table 2:** Lung function data of patients and controls

	**Controls, N = 16**	**ILD, N = 15**	**p**	**Emphysema, N = 16**	**p**	**Obese, N = 15**	**p**
Forced vital capacity (FVC) (L)	3.84 (0.94)	3.10 (1.08)	NS	2.95 (0.98)	NS	2.98 (0.69)	NS
FVC (% of pred.)	98.56 (12.00)	80.27 (15.61)	<0.005	76.75 (19.8)	0.003	91.13 (14.39)	NS
Forced expiratory volume in 1 s (FEV1 (L)	2.93 (0.78)	2.46 (0.84)	NS	1.24 (0.56)	<0.001	2.36 (0.55)	NS
FEV1 (% of pred.)	94.19 (15.58)	79.40 (14.49)	NS	39.81 (15.19)	<0.001	90.4 (16.27)	NS
FEV1/FVC (%)	0.76 (0.07)	0.80 (0.08)	NS	0.42 (0.11)	<0.001	0.80 (0.05)	NS
FEV1/FVC% (% of pred.)	94.38 (8.96)	99.67 (10.70)	NS	52.38 (13.80)	<0.001	99.6 (7.13)	NS
Peak expiratory flow (PEF) (L/s)	7.80 (1.73)	7.43 (2.84)	NS	3.43 (1.58)	<0.001	6.6 (1.38)	NS
PEF (% of pred.)	93.44 (16.08)	91.53 (24.56)	NS	41.56 (18.86)	<0.001	95.1 (21.09)	NS
MEF50 (% of pred.)	73.38 (31.71)	81.60 (29.71)	NS	12.25 (8.44)	<0.001	73.9 (26.2)	NS
MEF25 (% of pred.)	95.19 (52.52)	94.07 (51.77)	NS	23.77 (14.62)	<0.001	88.07 (30.92)	NS
Rtot (% of pred.)	129.44 (37.02)	168.27 (60.32)	NS	327.02 (176.1)	0.003	149.0 (36.8)	NS
sGtot (% of pred.)	90.75 (29.48)	98.53 (48.37)	NS	31.81 (16.25)	<0.001	81.13 (24.1)	NS
FRCpleth (Functional recidual capacity by plethysmograph (L)	3.86 (0.60)	2.77 (0.73)	<0.001	5.17 (1.13)	NS	2.46 (0.41)	<0.001
FRCpleth (% of pred.)	104.62 (14.14)	77.07 (15.70)	<0.001	129.2 (29.2)	0.007	89.5 (11.4)	NS
TLC (Total lung capacity) (L)	6.88 (1.18)	5.12 (1.32)	<0.001	7.38 (1.34)	NS	5.43 (0.89)	<0.005
TLC (% of pred.)	102.94 (12.08)	78.67 (13.66)	<0.001	111.23 (14.00)	NS	96.9 (8.79)	NS
RV (residual capacity) (L)	2.49 (0.37)	1.8 (0.33)	<0.001	3.86 (0.97)	0.002	2.02 (0.46)	NS
RV (% of pred.)	97.1 (16.2)	73.67 (12.3)	<0.001	150.8 (48.71)	0.004	95.2 (17.2)	NS
ERV (Expiratory reserve volume) (L)	1.37 (0.63)	0.97 (0.47)	NS	1.47 (0.56)	NS	0.47 (0.33)	<0.001
ERV (% of pred.)	121.4 (45.7)	85.8 (33.0)	NS	102.8 (36.1)	NS	71.3 (42.3)	NS
Trapped air (TLCb – TLC He) (L)	0.82 (0.36)	0.82 (0.38)	NS	2.08 (1.16)	0.004	0.71 (0.37)	NS
DLCOc (Single-breath diffusing capacity for carbon monoxide) (% of pred.)	93.9 (11.3)	51.1 (15.1)	<0.001	38.1 (16.9)	<0.001	85.9 (12.7)	NS
DLCOc/VA (Specific diffusing capacity) (% of pred.)	97.9 (8.4)	73.1 (15.3)	<0.001	45.3 (16.9)	<0.001	97.5 (15.8)	NS

Conventional spirometric data; results from constant volume body plethysmography and diffusing capacity measurements are given, ((Mean (SD)). The p-values indicate comparisons between controls and patients with pulmonary disease or obesity in non-paired *t*-test. Adjustments of t-tests have been performed as indicated in the Methods section. Bonferroni-corrected level of significance is p < 0.001. Pred. = predicted.

**Figure 1:**
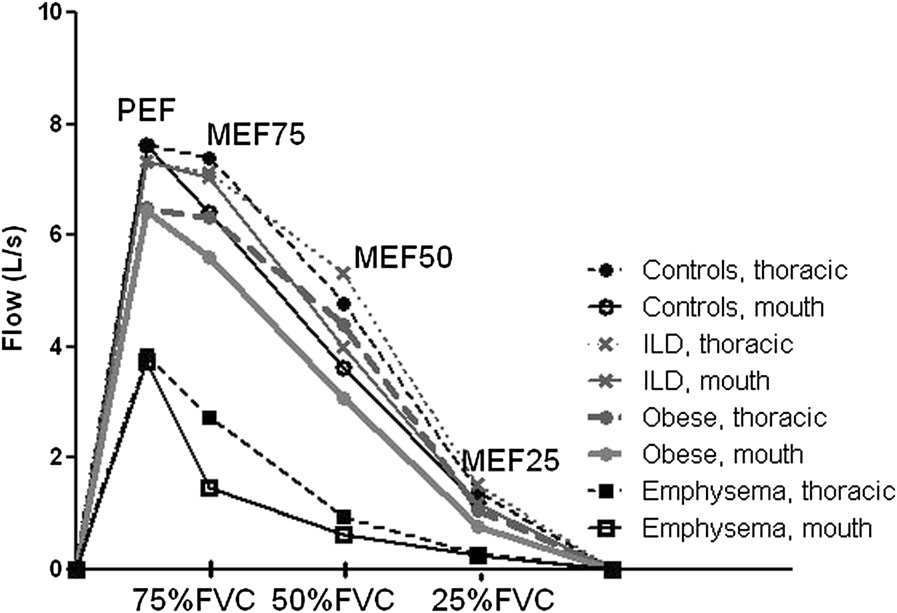
**Compressed (mouth) and compression-free (thoracic) flow values at different expiratory flow levels.** Compressed (mouth flow) and uncompressed airflow (thoracic flow) during forced expiration at peak expiratory flow level (PEF) and at different levels of expired volume (MEF75, MEF50 and MEF25).

A schematic presentation of gas flows during forced expiration at the thorax and at the mouth is provided in Figure 1, enabling the differences in gas compression for each of the four groups to be visualized. The quantitative results are given in Table 3. In all groups, a significant difference between thoracic and mouth flows was found at MEF50 (Table 3). In controls, a significant difference was measured also at MEF75, in emphysema at PEF and MEF75 and in obese subjects at MEF75 and as the only group additionally at MEF25.

**Table 3:** Gas compression at different levels of forced expiration according to patient group

	**Controls (N = 16) (L/s)**	**M, RD p-value**	**ILD (N = 15) (L/s)**	**M, RD p-value**	**Emphysema (N = 16) (L/s)**	**M, RD p-value**	**Obese (N = 15) (L/s)**	**M, RD p-value**
PEF mouth	7.62 (1.95) (5.0 – 11.2)	0.013 (-0.1 – 0.5)	7.33 (2.41) (3.2 – 12.4)	- 0.007 (-0.02 – 0.2)	3.73 (1.82) (1.9 – 7.6)	0.11 (-0.25 – 0.46)	6.45 (1.01) (5.1 – 8.3)	0.03 (0 – 0.21)
		NS				0.012		NS
				NS				
PEF thoracic	7.64 (1.96) (5.0 – 11.4)		7.33 (2.41) (3.2 – 12.4)		3.84 (1.85) (2.0 – 7.7)		6.48 (1.01) (5.1 – 8.3)	
MEF75 mouth	6.40 (1.74) (4.4 – 9.9)	1.03 (-0.14 –3.55)	7.05 (2.44) (3.2 – 12.3)	0.073 (-0.85 – 2.15)	1.45 (1.13) (0.3 – 3.6)	1.26 (0.0 – 3.89)	5.6 (1.11) (4.0 – 8.3)	0.75 (0 – 1.84)
MEF75 thoracic	7.42 (1.86) (4.9 – 11.0)	0.002	7.1 (2.35) (3.2 – 12.2)	NS	2.72 (1.93) (0.6 – 7.4)	0.001	6.31 (1.09) (4.8 – 8.3)	0.001
MEF50 mouth	3.62 (1.57) (1.5 – 8.1)	1.16 (-1,13 –3.33)	4.01 (1.12) (2.1 – 5.8)	1.5 (-0.63 – 3.78)	0.63 (0.37) (0.2 – 1.3)	0.3 (-0.13 – 1.39)	3.06 (1.01) (1.5 – 4.6)	1.33 (0.21 – 2.92)
MEF50 thoracic	4.78 (1.53) (2.8 – 8.2)	0.002	5.31 (2.00) (1.8 – 9.6)	<0.001	0.93 (0.59) (0.2 – 2.2)	0.01	4.40 (1.01) (3.3 – 7.0)	< 0.001
MEF25 mouth	1.18 (0.66) (0.5 – 3.0)	0.21 (-1.37 – 1.12)	1.18 (0.38) (0.5 – 2.0)	0.33 (-0.68 – 3.54)	0.24 (0.12) (0.1 – 0.4)	0.03 (-0.8 – 0.16)	0.77 (0.31) (0.4 – 1.2)	0.27 (-0.38 – 0.84)
MEF25 thoracic	1.38 (0.90) (0.3 – 4.2)	NS	1.51 (1.01) (0.4 – 4.6)	NS	0.27 (0.08) (0.1 – 0.4)	NS	1.05 (0.43) (0.4 – 1.9)	0.006

Compressed (mouth) and uncompressed (thoracic measurement) flow values (mean, SD and range) at different levels of forced expiration; at peak expiratory flow (PEF), maximal expiratory flow when 75% of forced vital capacity remains exhaled (MEF75), MEF 50 and MEF 25. Means (M) and ranges (RD) of the differences and the p-values of the paired *t*-test results between thoracic and mouth measurements in each group are presented.

**Figure 2:**
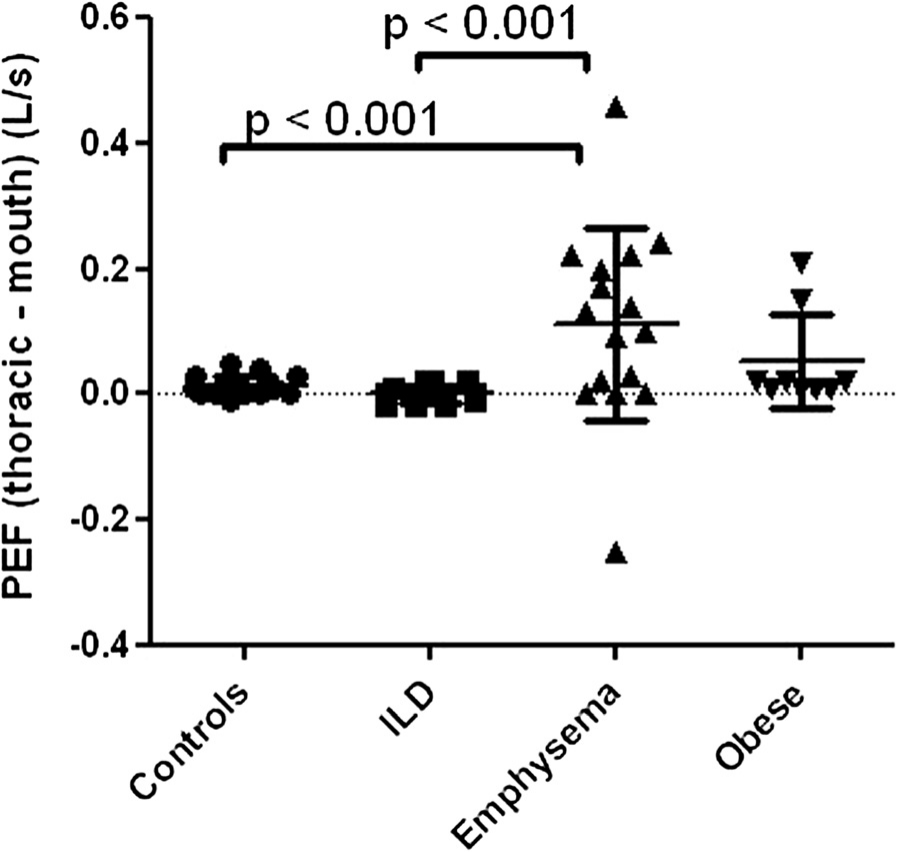
**Difference between thoracic and mouth flow at the level of PEF.** Level of significance after Bonferroni correction is p <0.008.

At the level of PEF, the difference between thoracic and mouth flows was significantly larger in patients with emphysema than in controls or patients with ILD (Figure 2).

**Figure 3:**
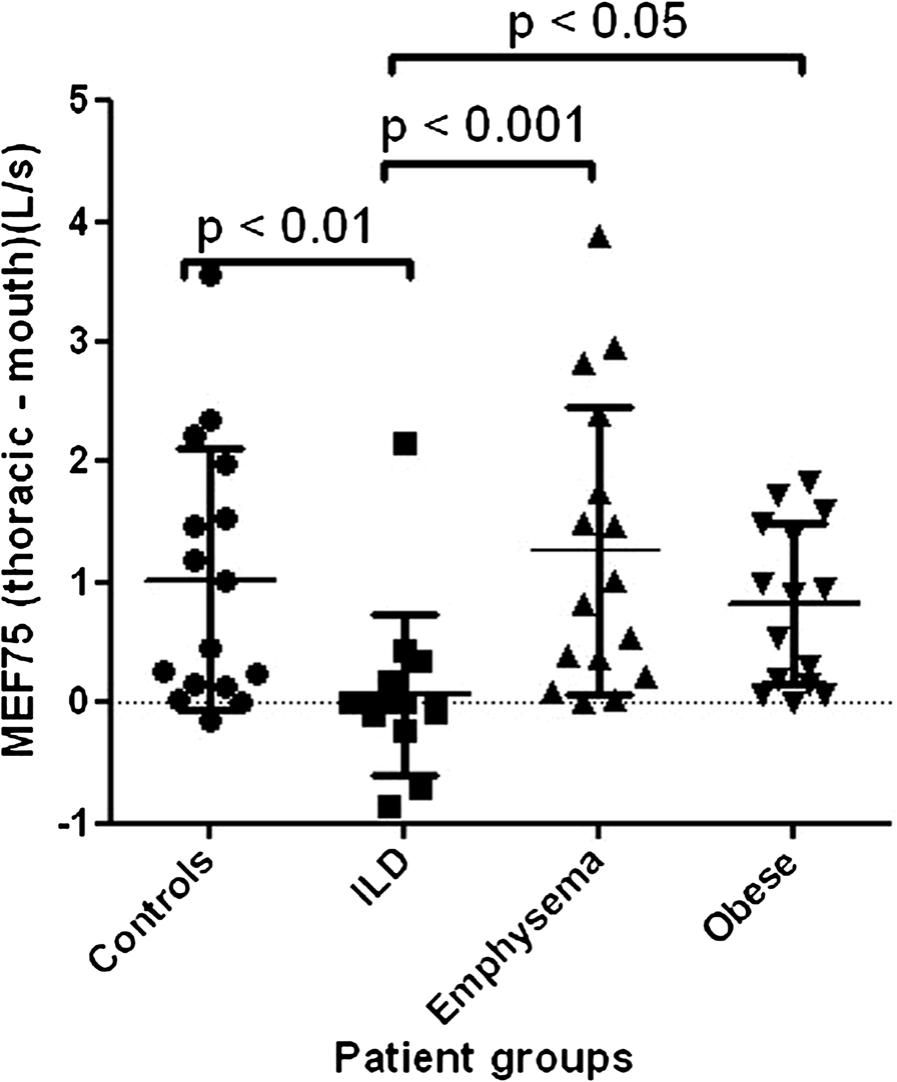
**Difference between thoracic and mouth flow at the level of MEF75.** Level of significance after Bonferroni correction is p <0.008.

When the patient groups were compared with each other, ILD showed the lowest difference between thoracic and mouth flows at MEF75 (Figure 3).

**Figure 4:**
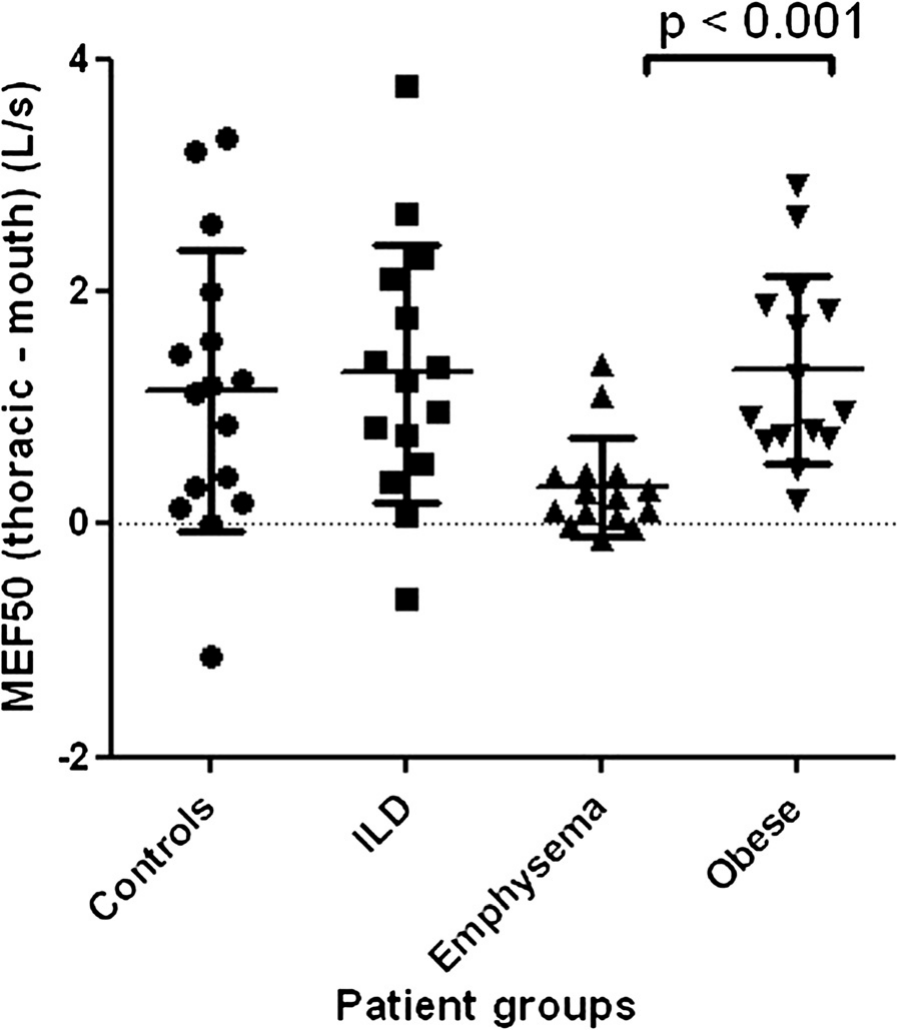
**Difference between thoracic and mouth flow at the level of MEF50.** Level of significance after Bonferroni correction is p <0.008.

**Figure 5:**
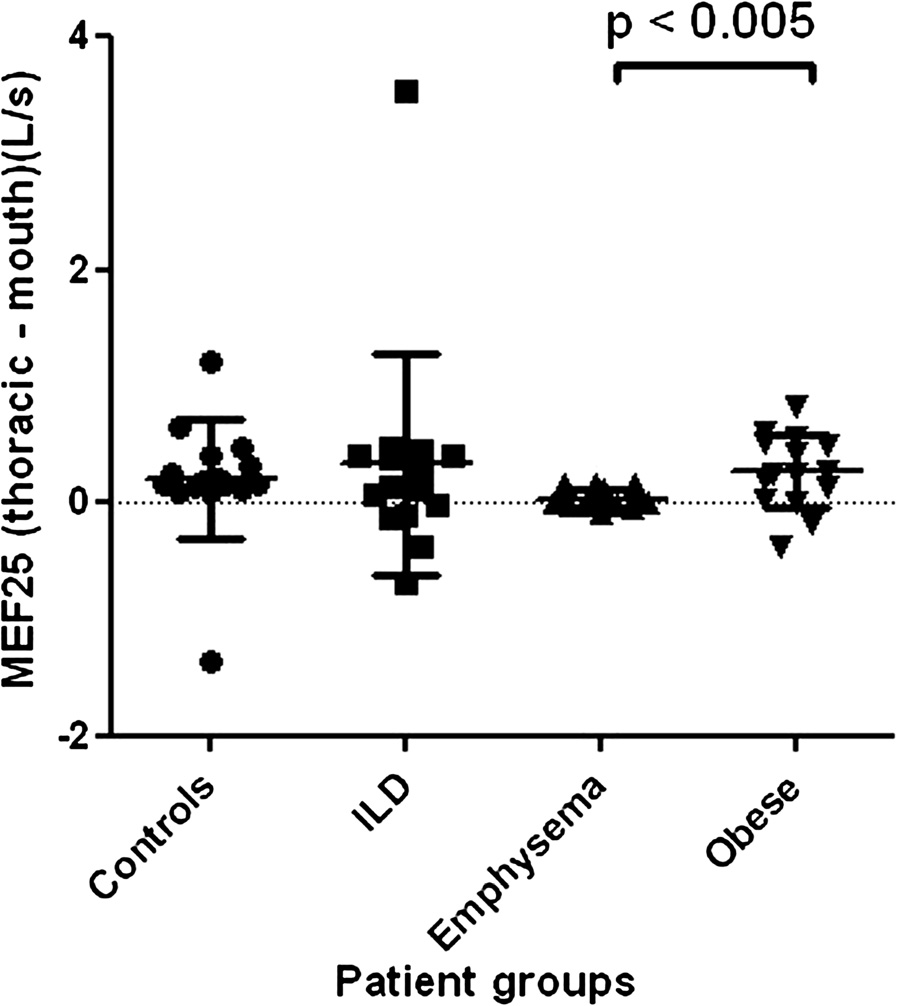
**Difference between thoracic and mouth flow at the level of MEF25.** Level of significance after Bonferroni correction is p <0.008.

At MEF50 and MEF25, the difference between thoracic and mouth flows was significantly larger in obese subjects than in patients with emphysema (Figures 4 and 5).

## Discussion

Significant differences between thoracic and mouth flows at forced expiration were observed in all patient groups in this study, at least at the MEF50 level. Results clearly indicate distortion of forced expiratory flow-volume curves measured by conventional spirometric methods. Some inter-individual variation and overlapping between study groups occurred. However, significant differences between thoracic and mouth flows can be explained by differencies in pulmonary mechanics, e.g. lung stiffness, obesity and decreased elastic recoil in emphysema which cause different airways’ resistances. Differences in flows were reduced in ILD at MEF75 and increased in emphysema at PEF and MEF75. In obesity, significant deviances were seen also at the level of MEF25.

Mead [19] developed the volume displacement body plethysmograph, and Jaeger and Otis [20] analysed volume displacement at the thorax and at the mouth and found the latter to be lower. This difference in volumes increased with increasing airway resistance, respiratory rate and lung volume. Later on, a flow plethysmograph was developed, easily changeable from constant volume mode to constant pressure mode (3).

In normal subjects, an increase of alveolar pressure in forced expiration parallels a compression of intrabronchial gas, upstream of the equal pressure point [1]. Forced inspiration, on the contrary, induces a decompression of alveolar gas. In bronchial obstruction, the intrabronchial gas compression in forced expiration has been reported to be increased [4,5,9], explained by the elevated airways resistance. Since in asthma the airways obstruction may vary, only patients with stable pulmonary diseases, without a significant bronchodilator response measured with spirometry, were included in the study.

The differences of thoracic and mouth flows in emphysema patients at the level of PEF were larger than in controls. PEF and MEF75 represent mostly characteristics of proximal airways, and MEF50 of more peripheral airways [12]. In emphysema, the support of bronchi is limited because of the emphysematic process increasing peripheral inhomogeneity and furthermore increasing airway resistance, which may explain the findings. Our results confirm earlier investigations in smokers [5] and COPD [11]. In emphysematic COPD, the peripheral obstructions typically affect the MEF50 and MEF25 values. In this study, MEF25 in patients with emphysema was very low because of elevated airway resistance. The differences of maximal expiratory flows measured by the thoracic and mouth pneumotachographs at this flow level were also elevated. In addition, in emphysematic COPD probably lowered muscle force in forced expiration may influence the expiratory flow values, although this component was not investigated here.

In obesity, the differences between thoracic and mouth flows also occurred at MEF25 level. Even though the difference at MEF25 was significant only in comparison with emphysema, this finding is remarkable. Decreased lung volume causes decreased airways calibers increasing airways resistance, which might be the source of this finding. The lungs have to work against increased intra-abdominal pressure (not measured here) [21-25]. Trapped gas and low expiratory reserve volume also contribute to unfavourable respiratory mechanics in obesity. For most of the obese subjects the specific conductance was in normal limits ≥46% of predicted value [14], which indicates that no bronchial obstruction was present in them.

The development of interstitial fibrosis decreases pulmonary compliance and is associated with inhomogeneous gas exchange. This study revealed that patients with ILD had unexpectedly low differences between thoracic and mouth flows at the beginning of maximal expiration. In ILD, elastic recoil forces are increased and airways stiffened, which decreases airways resistance. In a consequence dynamic airways compression in the large bronchi is decreased or abolished.

Especially in several patients with ILD, we observed an inverse behaviour between thoracic and mouth flows. It is difficult to explain the reasons to that finding. It could be possible that during forced expiration the upwards movement of the diaphragm might be attenuated due to stiff pulmonary tissue in ILD. Therefore, dynamic reduction of thoraco-abdominal volume during forced expiration may be decreased thus counteracting the pressure and volume displacements within the body box thereby decreasing the flow measured through the wall of body box (thoracic flow).

Another factor for the negative flow difference in ILD might be an instrumental error. Assuming that no phase shift occurs between measurements of mouth flow and thoracic flow and no instrumental errors exist, mouth flow exceeding thoracic flow would imply dilatation of some gas compartments in the lungs or elsewhere in the body during forced expiration.

As presented in Table 2 the total specific conductance (sGtot) was the highest in ILD compared with the other groups suggesting the least resistance. Based on the high specific conductance the changes in intrathoracic pressure, lung volume and air flow during forced expiration are, probably, more abrupt in ILD patients than in other patient groups. This might augment the effect of a possible phase shift between the measurements of mouth flow and thoracic flow. As presented by Goldman et al. [2] the volume change recorded by integrated flow across the plethysmograph wall is slower than that measured at mouth because of a temporary loss of volume during the initial decrease in plethysmographic air pressure. For comparison, in obstruction the time constant of the mouth flow could be high enough to fit with the compensation of the time constant of the box. In ILD, the time constant of the mouth flow is low and an incomplete correction of the box flow probably leads to negative differences.

The patient groups are well defined, and typical functional findings were made within each group. However, finding suitable patients for the study was difficult. The group of emphysema patients was on average older than other groups. Patients with pulmonary fibrosis were mostly on peroral steroids, therefore having tendency for overweight. Locating lean patients with ILD was thus challenging. In addition, it was challenging to find healthy elderly obese non-smoking men. In consequence, the BMI of patients with emphysema was lower than that of other patient groups and healthy subjects. The group of obese subjects mostly comprised women. A small, non-significant difference in age was also present between groups. To overcome these differences, comparisons between all participants were adjusted for these confounders, as indicated in the Methods section. However, the subgroups were rather homogenous and adjustments were not needed when comparisons within these groups were performed. Most of the variables were normally distributed. Because the available statistical program did not allow adjustment for non-parametric tests, parametric tests were used for all comparisons. The results in non-parametric and parametric testing did not differ significantly; therefore, no bias was likely caused by this restriction.

We have used a panting frequency of 0.5 Hz because the Finnish reference values for body plethysmography [14] have been determined for this panting frequency. Compared with medium or high panting frequencies, there might be slight differences in the functional residual capacity (FRC) values [26]. Originally, Jaeger and Otis (1964) [20] used spontaneous breathing and recommended avoidance of panting in body plethysmographic measurements.

## Conclusion

In conclusion, patient groups with distinct respiratory mechanics show different profiles of gas compression. All groups had elevated differences between thoracic and mouth flows in the mid-fraction of forced expiratory manoeuvre. The remarkable difference in thoracic and mouth flows found in emphysema is probably associated with inhomogeneity of the lung and decreased elastic forces increasing peripheral obstruction. The low difference between thoracic and mouth flows in ILD, can be explained by increased elastic forces decreasing airway resistance in the proximal airways.

Our results revealed remarkable levels of gas compression also in obesity in the middle and last parts of the maximal expiratory manoeuvre. These findings may help in the interpretation of conventional spirometric results, especially in obese subjects. A decrease in forced expiratory flow in non-smoking healthy obese subjects is more likely a result of gas compression than of real bronchial obstruction.

## Abbreviations

BMI: Body mass index; COPD: Chronic obstructive pulmonary disease; FEV1: Forced expiratory flow in one second; FRC: Functional residual capacity; FRCpleth: Functional recidual capacity measured by plethysmograph; FVC: Forced vital capacity; ILD: Intersitial lung disease; IPF: Idiopathic pulmonary fibrosis; PEF: Peak expiratory flow; MEF 75: MEF50, MEF25, Maximal instantaneous forced expiratory flow where 75%, 50% or 25% of FVC remains to be expired; NSIP: Nonspecific interstitial pneumonia; Rtot: Total resistance of airways; RV: Residual volume; sGtot: Total specific conductance of airways; TLC: Total lung capacity; VC: Vital capacity.

## Competing interests

The authors declare that they have no competing interests.

## Authors’ contributions

PP planned the study, collected patient data, calculated statistics and wrote the manuscript. UH participated in collecting patient data, especially on ILD patients, and contributed to writing the manuscript. TW participated in data collection and manuscript writing. H-JS contributed to writing the manuscript, assisting particularly with technical and mechanical points. AS participated in study design, patient collection and writing the manuscript. All authors read and approved the final manuscript.

## Authors’ information

PP, Chief Physician at the Unit of Clinical Physiology, Helsinki University Central Hospital.

UH, Senior Consultant at the Pulmonary Clinic, Helsinki University Central Hospital.

H-J S, engineer with a methodological background in lung function testing and physiological expertise.

AS, Professor of Clinical Physiology, Helsinki University.

## Pre-publication history

The pre-publication history for this paper can be accessed here:

http://www.biomedcentral.com/1471-2466/14/34/prepub
